# Residuos farmacéuticos domiciliarios en el medio ambiente: de la preocupación a la acción

**DOI:** 10.26633/RPSP.2021.155

**Published:** 2021-12-28

**Authors:** Berta Schulz-Bañares, Camila Sandoval-Cifuentes, Tamara Sandoval-Quijada, Claudio Müller-Ramírez

**Affiliations:** Departamento de Farmacia, Facultad de Farmacia, Universidad de Concepción Concepción Chile Departamento de Farmacia, Facultad de Farmacia, Universidad de Concepción, Concepción, Chile; Consultora independiente Concepción Chile Consultora independient, Concepción, Chile; Departamento de Farmacia, Facultad de Farmacia, Universidad de Concepción Concepción Chile Departamento de Farmacia, Facultad de Farmacia, Universidad de Concepción, Concepción, Chile; Departamento de Farmacia, Facultad de Farmacia, Universidad de Concepción, Concepción Chile Departamento de Farmacia, Facultad de Farmacia, Universidad de Concepción, Concepción, Chile

Al editor:

Los autores queremos hacer un llamado de atención a la comunidad sobre un tema de salud pública que silenciosamente afecta a todos los seres vivos. Se trata de la presencia de contaminantes emergentes (CE) (ej. plaguicidas, cosméticos, nanomateriales, fármacos, entre otros) en el medioambiente. Los CE se caracterizan por su persistencia, bioconcentración, bioacumulación, biomagnificación, y movilidad ambiental. Los efectos de los CE sobre la salud humana y otros seres vivos es motivo de estudio desde hace poco tiempo, por ello en latinoamérica mayormente no se dispone de normativa legal que regule este tema (1).

En países como, Brasil, Canadá, España, Francia, Inglaterra, Portugal y Uruguay se han realizado investigaciones que determinan la presencia de algunos CE en la entrada y salida de las plantas depuradoras de aguas servidas, demostrándose que no es posible su completa eliminación (1). El problema radica en que se desconoce su toxicidad y la de sus metabolitos, que en ocasiones es mayor. Entre los CE detectados en estos estudios, destacan los siguientes fármacos: carbamazepina, atenolol, sulfadiazina, paracetamol, eritromicina, ácido salicílico, diclofenaco, ibuprofeno, 17 β-estradiol, progesterona y levonorgestrel (1).

En cuanto a la presencia de fármacos en agua potable y el efecto que esto significa para la salud humana, se ha encontrado que sus concentraciones, en relación con dosis diarias admisibles, constituyen un riesgo despreciable, aunque hay consenso en la necesidad de futuros estudios de exposición crónica (2).

Asimismo, el posible impacto a largo plazo que puede causar el consumo de productos del mar contaminados con fármacos sigue siendo una preocupación, ya que se bioacumulan en ellos, sugiriéndose como la fuente más importante de estos contaminantes para el ser humano.

Por otro lado, existe evidencia que los fármacos presentes en el agua impactarían negativamente la fauna acuática. Es así como las hormonas naturales o sintéticas, son conocidas por causar gran riesgo para el ecosistema acuático, incluyendo la feminización de peces machos (3). De manera similar, los analgésicos influirían en la reducción en la tasa de alimentación, producirían impacto en marcadores bioquímicos y sobrevida, además de originar cambios en la respuesta inmune de algunos organismos marinos (3).

Si bien, el avance de la medicina en las últimas décadas ha impactado positivamente la salud de las personas, este escenario lleva consigo, entre otras cosas, el aumento de residuos farmacéuticos domiciliarios (RFD) asociado a las múltiples terapias farmacológicas disponibles (4). En Chile, los residuos peligrosos generados, tanto por la industria farmacéutica y los centros asistenciales (ej. hospitales, clínicas) están regulados en el “Reglamento sobre Manejo de Residuos de Establecimientos de Atención de Salud” aprobado por el Ministerio de Salud el año 2009, que en esencia busca evitar la generación de contaminación ambiental asociada, únicamente a sustancias con riesgo potencial y por ende la afectación de la salud de las personas.

A nivel nacional e internacional, el manejo de los RFD no cuenta con regulación. Esto, en concordancia con directrices avaladas por la Organización Mundial de la Salud (OMS) en el documento “Pharmaceuticals in drinking water” publicado en el año 2012 (5). Consecuentemente, los RFD son vertidos directamente en el alcantarillado o bien en la basura domiciliaria. Este escenario ha puesto de manifiesto gran preocupación en la comunidad científica, ya que los RFD, al ser CE presentes en el medioambiente, afectarían negativamente el ecosistema e indirectamente la salud de los seres humanos.

Bajo este escenario, se hace necesario fomentar acciones y estrategias de mitigación del problema, tales como los programas de devolución de RFD desarrollados e implementados en Estados Unidos, Ghana, Irlanda, Nueva Zelanda, Suecia, Uruguay, entre otros (6). Éstos consisten en recuperar RFD en puntos geográficos establecidos (6) y de esta manera sensibilizar a la comunidad sobre la importancia de elegir opciones respetuosas con el medio ambiente para desechar los RFD.

Solamente Australia adoptó una política pública para eliminar adecuadamente los fármacos a nivel nacional de forma gratuita, el llamado Retorno Nacional y Eliminación de medicamentos no deseados (NATURUM de sus siglas en inglés), que se implementó en 1998 y está disponible en todas las farmacias australianas (6).

Finalmente, el manejo de los RFD es una situación compleja de abordar, sobre todo si aproximadamente el 50% de los medicamentos, a nivel mundial, son prescritos inapropiadamente (7). Por esta razón, se debe realizar un esfuerzo conjunto de todos los actores involucrados en el ciclo de vida de los medicamentos, incluyendo autoridades gubernamentales, instituciones públicas y privadas, la academia, la comunidad científica y la población en general, para optimizar el uso racional de medicamentos y reducir la cantidad de RFD dispuestos en el medioambiente, ya que de esta manera se estaría reduciendo el deterioro ambiental y protegiendo la salud de las personas.

## Declaración.

Las opiniones expresadas en este manuscrito son responsabilidad de los autores y no reflejan necesariamente los criterios ni la política de la RPSP/PAJPH y/o de la OPS
